# Quantification of Endogenous Brain Tissue Displacement Imaging by Radiofrequency Ultrasound

**DOI:** 10.3390/diagnostics10020057

**Published:** 2020-01-21

**Authors:** Rytis Jurkonis, Monika Makūnaitė, Mindaugas Baranauskas, Arūnas Lukoševičius, Andrius Sakalauskas, Vaidas Matijošaitis, Daiva Rastenytė

**Affiliations:** 1 Biomedical Engineering Institute, Kaunas University of Technology, K. Baršausko Str. 59-455, LT-51423 Kaunas, Lithuania; monika.makunaite@ktu.edu (M.M.); m.baranauskas@ktu.lt (M.B.); arunas.lukosevicius@ktu.lt (A.L.); sakalauskas.andrius@yahoo.com (A.S.); 2 Department of Neurology, Lithuanian University of Health Sciences, A. Mickevičiaus Str. 9, LT-44307 Kaunas, Lithuania; vaidas.matijosaitis@lsmuni.lt (V.M.); Daiva.Rastenyte@lsmuni.lt (D.R.)

**Keywords:** brain, transcranial sonography, radiofrequency ultrasound, tissue displacements

## Abstract

The purpose of this paper is a quantification of displacement parameters used in the imaging of brain tissue endogenous motion using ultrasonic radiofrequency (RF) signals. In a preclinical study, an ultrasonic diagnostic system with RF output was equipped with dedicated signal processing software and subject head–ultrasonic transducer stabilization. This allowed the use of RF scanning frames for the calculation of micrometer-range displacements, excluding sonographer-induced motions. Analysis of quantitative displacement estimates in dynamical phantom experiments showed that displacements of 55 µm down to 2 µm were quantified as confident according to Pearson correlation between signal fragments (minimum *p* ≤ 0.001). The same algorithm and scanning hardware were used in experiments and clinical imaging which allows translating phantom results to Alzheimer’s disease patients and healthy elderly subjects as examples. The confident quantitative displacement waveforms of six in vivo heart-cycle episodes ranged from 8 µm up to 263 µm (Pearson correlation *p* ≤ 0.01). Displacement time sequences showed promising possibilities to evaluate the morphology of endogenous displacement signals at each point of the scanning plane, while displacement maps—regional distribution of displacement parameters—were essential for tissue characterization.

## 1. Introduction

Brain tissue pulsates during cardiac and breathing cycles [1]. Endogenous quasi-periodic pulsatility in the blood basin forces brain tissue to expand and relax by a fraction of a percent [2], and this small pulsation depends on the mechanical properties of the tissue [3]. Since the magnitude of displacement correlates with a stiffness of the medium [3], quantitative parameters of tissue displacement signals have the potential for diagnostic purposes. Invasive and noninvasive technologies make it possible to investigate intracranial pulsatility as well its relationship to neurological diseases, however, the validation of non-invasive methods depends on technical advances of pulsatility measurements and data analysis [4].

The small displacements of brain areas can be estimated using backscattered ultrasound (US) signals and tissue Doppler signal processing methods for a radiofrequency (RF) US. This allows the investigation of endogenous displacements of brain areas excited by respiratory and cardiac activity [1,2,5,6,7]. The popular version of this method was developed by Kucewicz et al. [2,5] and called tissue pulsatility imaging (TPI). Brain tissue pulsatility measured by TPI is closely related to changes in cerebral blood flow [2,5,6]. The most recent research [8,9,10] deals with the development of quantitative shear wave-based passive elastography. Galot et al. [8] and Brum et al. [9] investigated methods which allow the recovery of quantitative elasticity (shear modulus) distribution by using ultrafast US scanning and time-reversal techniques. Unfortunately, such tomographic time-reversal based techniques are computationally expensive and require dedicated hardware. To our knowledge, current non-targeted ultrasonic methods have a limitation when locating brain structures—none of the previous brain pulsatility studies were targeted to the quantitative mapping of specific brain structures, since they were applied for the estimation of global pulsatility of the brain. The aspects of methods’ immunity to external movements, the stabilizing techniques during transcranial echoscopy, and the technical potential of algorithms of quantitative tissue pulsatility detection are still of limited examination.

The purposes of the paper are to (a) describe an RF ultrasound-based approach capable of measuring micron-level displacements using phantom and in vivo experiments; and (b) demonstrate the feasibility of this approach in estimating tissue displacement caused by endogenous motion, such as cardiac pulsation, in particular brain structures.

The modeling of dynamic displacements using dedicated agar-based motion phantom is performed in the range of endogenous displacements found in vivo in the brain. Then, the in vivo scanning protocol is described, including developed means for stabilization of the head-transducer position, since the high sensitivity of the system and requirement of reliability without excessive averaging requires the acquisition of especially reliable RF US signals.

The confidence of detected displacements is an issue of attention in this study. For confidence quantification in displacement map of brain tissue, analysis is focused on the cardiovascular pulsing as a repeatable endogenous source. The processing algorithm of obtained RF US signals is developed for quantitative imaging (mapping) of endogenous displacements with possibly high resolution and confidence on the map. The performance was demonstrated by a quantitative comparison of pre-clinical tests and clinical results of endogenous brain displacement of the hippocampus cross-sectional area of brains with Alzheimer’s disease and healthy elderly brains. The confidence map of repeatable waveforms is an example of possible diagnostic differentiation of targeted brain structures using quantitative and confident micrometer-scale displacement parameters.

## 2. Materials and Methods

### 2.1. Modeling of Displacements

**Figure 1:**
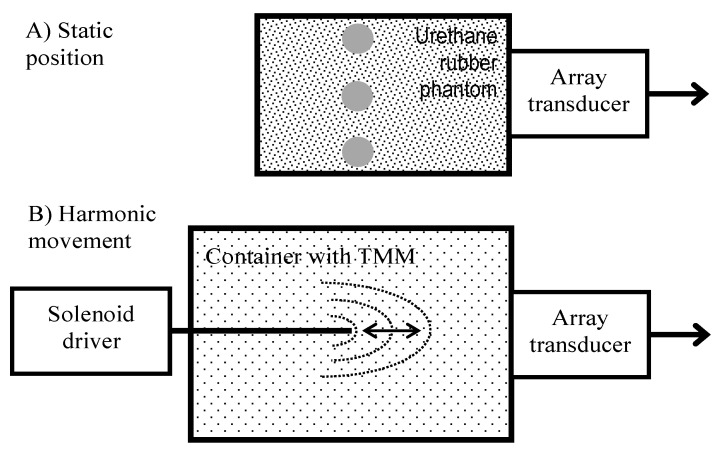
Structures of phantoms used in the evaluation of displacement detection system: (**A**) static structure (Model 532A); (**B**) structure with a harmonic motion phantom with displacement field (concentric dotted lines) excited in tissue-mimicking material (TMM).

Pre-clinical modeling of displacements was elaborated with the help of static structure phantom and dynamic motion phantom of harmonic displacements of known frequency (see Figure 1). Contrast resolution phantom (Model 532A, ATS Laboratories Inc., Bridgeport, CT, USA) embedded in urethane rubber structure was used as a motionless structure. The RF data for estimation of the standard deviation of position detection were obtained by scanning the cylindrical (D = 8 mm) hyperechoic (+12 dB) structure. The motionless structure was required to verify means for the stabilization of the head-transducer position. To verify the quantitative conformity of detected displacements, we used a dedicated harmonically-excited motion phantom. The agarose gel was used as tissue-mimicking material (TMM). The background of the phantom was manufactured by mixing 5 g/L agar concentration in distilled water. The predicted [11] Young modulus was 7 kPa. To obtain adequate scattering we dispersed 0.3 g of graphite powder in 1000 mL of agar mixture. The preparation of TMM is described in [12]. A wooden rod (2 mm in diameter) was embedded in the TMM. This rod was fixed to a solenoid actuator, inducing a linear push–pull motion at a defined frequency, which was set by the generator. Rod displacements caused a strain in an adjacent region of agarose gel. The photo of motion phantom is provided in Figure S2.

As the source of the harmonic motion, we used a solenoid electromagnetic driver constructed in our laboratory (see Figure 1B). The stator was a cylindrical permanent magnet. The solenoid coil a core made of a ferromagnetic rod. The coil was supported by elastic plates, allowing its oscillation. The coil had a resistance of 1.5 kΩ and inductance of 1.6 H. The extension of the coil core was fixed to a wooden rod and embedded about 90 mm in the TMM material. The TMM holding container had thin walls of plastic and this plastic permitted ultrasound waves to pass into the TMM. The phased array transducer was held by a dedicated positioning system (see Supplementary Materials) in acoustic contact with the container wall. The direction of rod motion was aligned along the centerline of the B-scan sector, so the motion towards and away from the phased array transducer was easily observed. The coil of the solenoid was excited by the harmonic waveform generator Rigol DG5252. Frequencies were of 2 Hz, 4 Hz, and 8 Hz, and the double amplitude was varied, with the setting appropriate for the generator output. It was assumed that the moving rod excited TMM was repeatable at a frequency of solenoid excitation, and motion magnitude was distributed non-uniformly in TMM. The excitation amplitude on terminals of coil were measured with a handheld HDS1022M-N oscilloscope (Fujian Ltd., Zhangzhou, China) during all trials. By controlling with oscilloscope measurements we assured excitation increments of 0.5 V peak-to-peak, in the range from 0.5 Vpp up to 3 Vpp. The point reflector was scanned with pre-set imaging parameters, the same as in clinical scanning. The acquired sequences of RF data were used to verify the motion-tracking algorithm by induced displacements of TMM. Verification included system reliability in the detection of time repeatable displacement waveforms.

### 2.2. Scanning Protocol

A research dedicated ultrasound scanner Ultrasonix Sonix Touch (Analogic Ultrasound, Richmond, BC, Canada), equipped with a phased array sector probe (SA 4–2, 64 acoustic elements), was used for all data collections in the study. The main parameters of ultrasonic scanning and RF signal digitization were as follows: sampling frequency—40 MHz, analog-to-digital converterresolution—16 bits, number of post-beamformed scanning lines—131, angle of phased array transducer sector—60°, echoscopy depth—11 cm, frequency of ultrasound waves—2.5 Hz, frame rate—45 Hz, transmit focal depth—7 cm. The sound speed in the scanner and for the processing of RF data was set to 1540 m/s. The sequences for recorded B scans (259 frames in total) and raw B-scan forming RF signals were acquired and stored for off-line analysis.

Before starting RF acquisition from the subject, we considered possible external movements induced by the sonographer and environmental vibrations. The sonographer (VM) and researchers (MM, RJ) hand-induced artefacts and found peak-to-peak magnitudes in the range of 19 µm to 36 µm. Calculating the root mean square (RMS) of motion signal of hand artefacts, we found a range from 3.4 µm to 6.6 µm. Therefore, stabilization was needed. In the room of the clinical department, we tested the developed holding system of an ultrasound transducer on the static phantom. Urethane rubber-based phantom ATS Model 532A, as motion-less structure, was used instead of a patient. The phantom was placed on the couch, where the head is located when scanning patients. We found peak-to-peak environment-induced artefacts to be up to 2 µm only when using the transducer holding system proposed. These spiking artefacts were found to be related to the vibration of the floor in the sonography room if there was some movement in the room. These trials raised the issue of a non-disturbed room during the acquisition of RF ultrasound signals. During acquisition, no walking or door closing was allowed; these precautions were included in our scanning protocol.

Ethical Approval: All procedures performed in studies involving human participants were in accordance with the ethical standards of the institutional and/or national research committee and with the 1964 Helsinki declaration and its later amendments or comparable ethical standards. Informed Consent: Informed consent was obtained from individual participant included in the study.

This study was approved by the Kaunas Region Biomedical Research Ethics Committee (19 December 2017, No. BE-2-728, Kaunas, Lithuania). Participants provided written consent to participate in the study and allowed the usage of the obtained B-scan images and RF signals under the principle of confidentiality. 

Transcranial sonography was performed on a supine subject. The holder system of the phased array transducer and holder of the subject’s head were implemented. Both holders made it possible to acquire endogenous brain motion, minimizing the influence of external movements. A description of the holder system is provided in the Supplementary Materials. B-scan images and RF signals of the brain were obtained through the temporal window. The subject was instructed before sonography to keep their body and head motionless when requested by the sonographer. After adjustment of the scanning beam to the hippocampus cross-sectional view, the transducer was fixed by tightening the spherical bearing (see Figure S1). The subject was asked to concentrate maximum attention and keep their head stable when the ultrasound B-scan images and RF data were collected. For this purpose, the sonographer informed subject by counting loudly to six–eight, so the subject could understand that special patience is required to get motionless recordings. The sonographer touched neither the subject nor the array transducer or couch on which the subject was supine. The sequence of RF data was acquired into cine-loop on temporal memory. On the best B-scan image the structure of the hippocampus cross-section was annotated, drawing the line around the region of interest (ROI). The annotated structure, as a picture, and the whole sequence of RF data (356 s right before annotated frame) were stored digitally on the hard disc of the ultrasound scanner. Ultrasound data were transferred to the desk computer for further off-line analysis in MATLAB.

### 2.3. RF Signal Processing Algorithm

The same ultrasonic RF signal processing algorithm was used both for phantom-based and further pre-clinical trials to compare quantitative results. The tissue displacements along the beamline were estimated by combining the cross-correlation function, calculated between the obtained RF signal segments, and parabolic interpolation [13] of the peak of the function. The cross-correlation lag between the segments at adjacent scanning frames *k* and *k* + 1 represents the inter-frame estimate of displacement magnitude. The correlation peak, in the presented approach, was estimated as follows:

(1) $$C_{k}\left[m\right]=\underset{-T\leq z\leq T}{\max}\left[{ℑ}^{-1}\left\{ℑ\left[w\left[z\right]\cdot D_{k}^{*}\left[z\right]\right]\cdot ℑ\left[w\left[z\right]\cdot D_{k+1}\left[z\right]\right]\right\}\right]\;,$$

where *D* is the RF signal segment at a certain scanning line and scanning depth, ℑ is the fast Fourier transform, acquisition instance (frame number) *k* = 1…*K*, * the asterisk denotes the complex conjugate. The empirical threshold value *T* was set at ±150 μm, *w* is the Hamming window function, *z* = 1…*Z*, *Z* is the number of samples of RF the signal segment (1.85 mm of six periods of US wave) used for the assessment of displacements, *m* is the correlation lag at the peak of the function. The threshold served for the prevention of false peaks, which occur when a secondary correlation peak exceeds the primary peak. The threshold was set after analyzing the outcomes of the algorithm—the estimated displacement signals. The tapering function was applied to suppress the leakage in the frequency domain and to increase the probability of the right correlation peak detection.

In the next step, parabolic interpolation of the correlation peak was applied to achieve sub-sample resolution of the displacement’s estimates. Parabolic interpolation is frequently used because it is efficient and simple to implement [13]. The peak-offset estimate of the inter-frame displacement *ID* was defined as follows:

(2) $$ID\left[k\right]=\Delta d\cdot \;\left(\frac{C_{k}\left[m-1\right]-C_{k}\left[m+1\right]}{2\cdot \left(C_{k}\left[m-1\right]-2\cdot C_{k}\left[m\right]+C_{k}\left[m+1\right]\right)}+C_{k}\left[m\right]\right),$$

where *C_k_*[*m* − 1] and *C_k_*[*m* + 1] are the nearest neighbors of the largest sample of cross-correlation function (*C_k_*[*m*]), Δ*d* is the sampling period (Δ*d* = 19.25 µm), *k* is the acquisition instance (frame number), *k* = 1…K. The signal of detected displacements *d* relative to transducer was calculated by the integral (cumulative sum) of the inter-frame displacement signal *ID*.

Displacement intensity was assessed by calculating the root mean square of the inter-frame displacement signals at each spatial point:

(3) $$d_{rms}\left[p\right]=\sqrt{\frac{1}{K}\cdot \sum_{k=1}^{K}ID_{p}^{2}\left[k\right]},$$

where *p* = 1…*P*, *P* is the number of RF data points in the B-scan, acquisition instance (frame number) *k* = 1…*K*, *ID_p_*[*k*] is the inter-frame displacement signal at the *p*-th spatial point. A calculated array of displacement intensities can be mapped on the grid of B-scan image.

The basis of all displacement assessments was the absolute values calculated at each point of the scanning plane. The straightforward approach is to construct a map of certain displacement parameters. However, for the diagnostic purposes and quantitative comparison of displacements in certain brain tissue regions of interest (ROI), other approaches based on the averaging of displacements in space and time are applicable. One of them is to evaluate the amplitude parameters of displacement signals from the entire ROI over certain time intervals. Averaging could improve the signal-to-noise ratio of the weak displacement estimates in the range of a few tens of micrometers. Also, for this purpose signal filtering targeting the hearth rhythm and aiming to eliminate respiration and autoregulation-related motions could be applied, but this, of course, could filter out displacement components with clinical significance, therefore it should only be applied if necessary. In the simplest case of amplitude parametrization, the first stage of the algorithm is averaging of the displacement signals obtained in the ROI. The ROIs were defined manually by the experienced neurosonographer and in our study, it was a cross-sectional area of the hippocampus. As an example, a B-scan image together with an outlined cross-section of the hippocampus is presented in Figure 7A. As a result, the average displacement signal of the outlined ROI was obtained. Averaging in space and time was done in order to increase the signal-to-noise ratio. The average displacement signal was expressed as follows:

(4) $$\bar{d\left[k\right]}=\frac{1}{P}\cdot \sum_{p=1}^{P}d_{p}\left[k\right],$$

where *p* = 1…*P*, *P* is the number of RF data points in ROI outlined by the neurosonographer, *d_p_*[*k*] is the displacement signal at the *p*-th position in ROI. The same averaging calculation was applied for inter-frame displacement *ID_p_*[*k*] as for accumulated displacement *d_p_*[*k*]. Then, the obtained displacement signal was high-pass filtered (cut-off frequency = 0.75 Hz) to focus on displacements related to heart rate and to filter out slow movements arising due to respiration. Then, other time averaging of confidence assessment could be applied, using the periodicity of endogenous cardiovascular excitation.

### 2.4. Assessment of Displacement Confidence

Since endogenous displacements are extremely small, the question of assessment confidence is essential. Here, we present a method which restricts the possible displacement values, employing specificity of endogenous brain tissue motion related to the heart rhythm.

The confidence of the *p*-th spatial point’s filtered (0.75 Hz high-pass) accumulated displacement signal *d_fltr_* was evaluated by motion pattern repeatability at the heart rhythm:

- The peak frequency *f_peak_*[*p*] of every *d^ftlr^*^[*p*]^ was identified by spectral analysis using a fast Fourier transform algorithm. A new subset, *A*, was formed from points that satisfied the condition *f_peak_*[*p*] ∈ [40, 125] in beats per minute units (i.e., from ~0.67 Hz to ~2.08 Hz):
- The *dominant frequency f_dmn_* was determined as the most frequent value in *f_peak_* of points from subset A. A new, narrower, subset B was formed only from points that satisfied the condition *f_peak_*[*p*] *= f_dmn_*;
- The *d^ftlr^* signals were averaged only from subset *B* points according to Equation (4);
- The template signal *d_temp_* was formed by taking part of *d_B_* signal that belongs to the [–0.3, 0.3] seconds window relative to *t_ref_*;
- Time at the extreme value of the *d_B_* derivative—derivative of the *d* signal at frame *k*, calculated as (*d_k_*_+ 1_ *− d*_k − 1_)/2 × 45—*d_temp_* was checked to have a positive peak and negative peak, and to have at least 0.05 s outside these peaks, otherwise *t_ref_* (and accordingly *d_temp_*) was changed to time of the next extreme value of the *d_B_* derivative;
- The waveforms similar to *d_temp_* were automatically detected using the normalized cross-correlation function [14]; the waveforms were assumed similar if the normalized correlation coefficient was ≥0.8. The time windows of these similar waveforms were identified as epochs;
- The same epochs were used to cut *d^ftlr^*[*p*] signals into segments for every spatial point separately (also for points not belonging to subset *A*). Pearson correlation *r*[*p*] was calculated between each pair of segments that belonged to the same spatial point. For example, for *N* epochs, *N ×* (*N* − 1)/2 correlations were calculated per one spatial point. The *p*-th point was considered confident if all Pearson correlation *r*[*p*] > 0 and all correlation *p*-values were ≤0.01 for the *p*-th spatial point.

### 2.5. Pre-Clinical Verification

**Figure 2:**
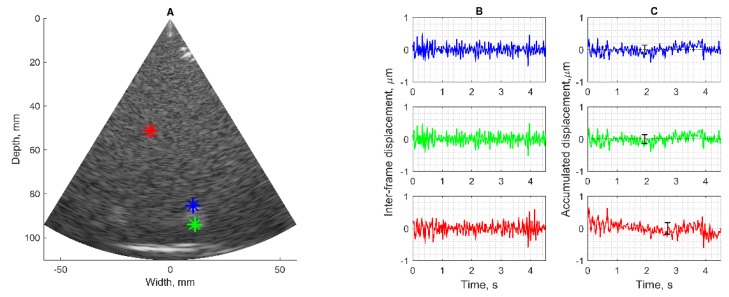
Results of the detection algorithm evaluation of targeted radiofrequency (RF) signals from the static phantom (Model 532A): (**A**) B-scan of the phantom structure with targets of interest; (**B**) the detected positions of static targets presented in the form of inter-frame displacements; (**C**) the calculated variability of static target positions or displacements relative to the transducer. Mean and standard deviation calculated from *n* = 21, error bar illustrates standard deviation up to 0.2 µm.

The motion-tracking algorithm was evaluated on RF data from physical models of static position and harmonic motion phantom. Echoscopy of phantoms with the use of the phased array transducer holding system for the stabilization of artefact movements was applied to register ultrasonic RF signals. RF data were recorded in the room of the clinical department, so considering the clinical environment with possible external vibrations. Targets of interest were chosen by observing B-scan images of the phantom (Model 532A). Targets chosen for detection in the static urethane rubber-based phantom, shown with colored star signs in Figure 2A, were marked by observing B-scan images of the phantom. One target was selected in the tissue-mimicking pattern (star sign of red color) the other two targets were chosen on edges of hyperechoic inclusion (star sign of blue and green color). The detection algorithm used is described in the Section 2.3. In Figure 2B, the detected positions of three targets are presented as inter-frame displacement signals in the time span of more than 4 s (line color corresponds to the color of the target). The inter-frame displacement signal represents displacements detected in the RF signals of neighboring frames of echoscopy. The signal of the detected position relative to the transducer was calculated by the integral (cumulative sum) of inter-frame displacements. In the case of the static phantom, the signal of displacement (see Figure 2C) represents the magnitude of random error of position detection.

## 3. Results

### 3.1. Mapping of Displacement Parameters in Phantom

**Figure 3:**
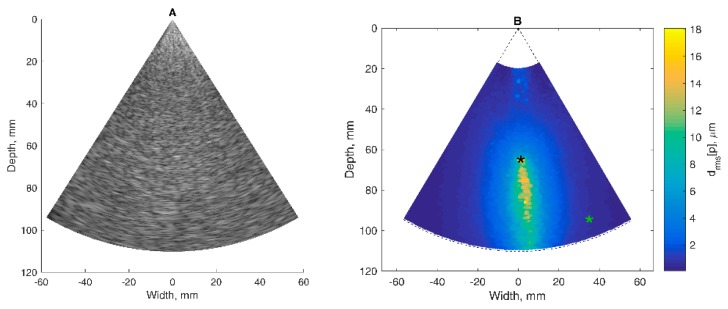

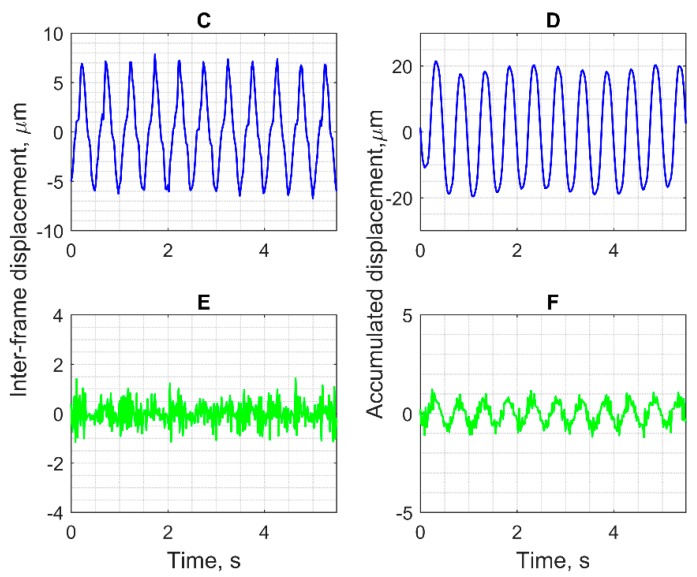
Results of the detection algorithm evaluation on targeted RF signals from the harmonic motion phantom: (**A**) B-mode ultrasound image of the phantom with the TMM; (**B**) map of motion: root mean square of the accumulated displacement signal *d_rms_*[*p*] represented in color scale. asterisks indicates the points in which weak (green asterisk) and intense (black asterisk) displacement signals were estimated; (**C**,**E**) displacement signals at weak and intense movements locations represented in the form of inter-frame displacements; (**D**,**F**) displacement signals at weak and intense movement locations represented in the form of accumulated displacements.

The results of the detection algorithm evaluation on targeted RF signals from the harmonic motion phantom are presented in Figure 3. The detected waveforms of 2 Hz harmonic displacements induced in TMM are provided in Figure 3C–F. The large part of the map represents relatively big displacements by the wooden rod, which entered TMM from the bottom of the maps and is seen in the center part of the B scan (*d_rms_* up to 20 µm in Figure 3B). The moving rod also induced vanishing displacements in the adjacent regions of TMM. The end-tip of the rod was located at 65 mm depth (black star in Figure 3B). The displacement waveform at this point is presented in Figure 3D. About 20 µm in magnitude, the largest displacements (yellow-colored in Figure 3B) were observed on the rod. The further away from the rod axis the weaker the detected displacements. More than 30 mm away from the rod (green star in Figure 3B) the displacements were less than 1 µm in magnitude and degraded by noise (Figure 3F). So, it was assumed that the side parts of the map were noise-corrupted, because they were far from the excitation axis.

**Figure 4:**
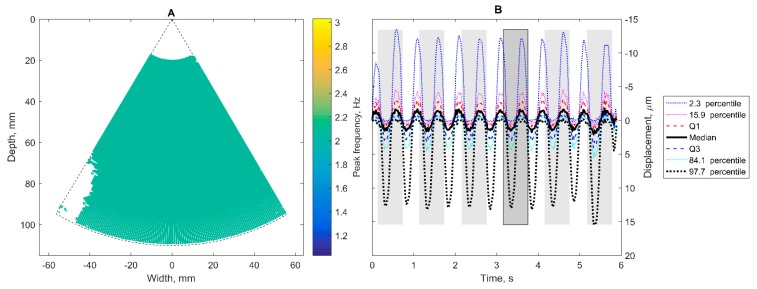
Oscillations of accumulated displacements. at dominant frequency of 2 Hz in dynamic phantom: (**A**) areas where 2 Hz frequency was determined as the peak frequency in spectral analysis; (**B**) epochs (grey rectangles) are time windows of similar waveforms in the average signal of points with dominant frequency; similarity detected by reference-template matching technique; here six epochs were identified.

The dominant frequency (here 2 Hz), determined as the most frequent value of peak frequency from points with spectral peak frequency within 0.75–5 Hz, is mapped in Figure 4A. The colored map provides an overview of points of only 2 Hz-related displacements. The uncolored locations in Figure 4A (mostly at the left corner) are presumably corrupted by noise. For further confidence evaluation by Pearson correlation, six 600 ms epochs were selected as similar waveforms in the average signal of points with dominant frequency (Figure 4B). The range of displacements detected in the motion field is presented with quartiles and percentiles in Figure 4B too.

**Figure 5:**
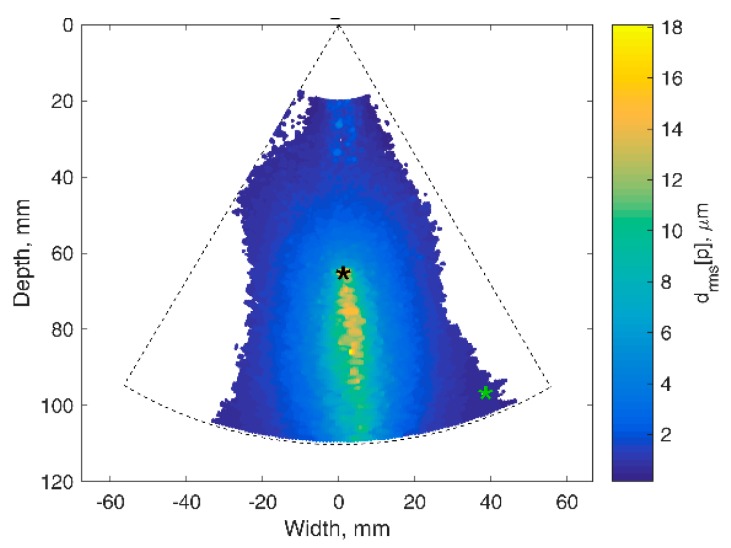
Confidence mapping of displacements in harmonic excited TMM: locations of confident (*p* ≤ 0.001) displacements root mean square in color scale. Asterisks in the graph indicates the points in which weak (green asterisk) and intense (black asterisk) displacement signals were estimated.

Confidence mapping of displacements in harmonic-excited TMM is shown in Figure 5. The large part of the map represents relatively small displacements (rms up to 10 µm). In the center part of TMM, the displacement field spreads from the excitation axis (wooden rod). So, the largest displacements (rms more than 10 µm) were observed near the rod. The further away from the rod axis, the weaker displacements. More than 30 mm away from rod the displacement rms were less than 2 µm, and further, the detected waveforms appeared non-repeatable and therefore non-confident. It was assumed the side parts of the map were noise-corrupted and therefore they were not depicted with color scale.

**Figure 6:**
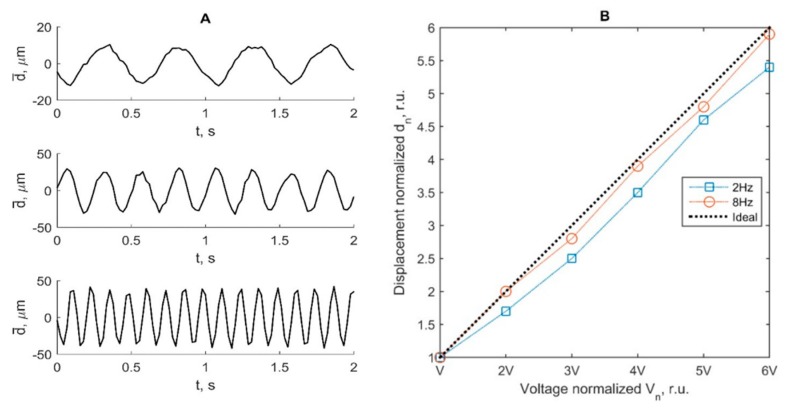
(**A**) The detected displacement signals for the 2 Hz (**upper**), 4 Hz (**middle**), 8 Hz (**lowest**) solenoid excitation frequencies; (**B**) detected displacements resulting from proportional changes in excitation amplitude of the solenoid. The dashed line represents the ideal theoretical case of detection. Excitation peak-to-peak voltages were 0.5 V, 1 V, 1.5 V, 2 V, 2.5 V, and 3 V at frequencies of 2 Hz and 8 Hz.

The dynamic resolution of the motion tracking algorithm was verified using motion phantom with known, controllable, frequencies and amplitudes. In Figure 6, the detected signals from simulated motion on 2 Hz, 4 Hz, and 8 Hz frequencies of solenoid excitation are shown. The detected displacement signals for these frequencies are presented in Figure 6A. The solenoid excitation at 2 Hz, 4 Hz, and 8 Hz frequencies resulted in displacements of peak-to-peak amplitude in the range from 3 to 124 µm. At two frequencies (2 Hz and 8 Hz), linearity errors not more than 17% and 9%, respectively, were obtained (see Figure 6B). The proportion between changes in excitation magnitude and detected displacement was kept, as well as a harmonic signal pattern at all frequencies.

### 3.2. Mapping of Intracranial Displacement Parameters

**Figure 7:**
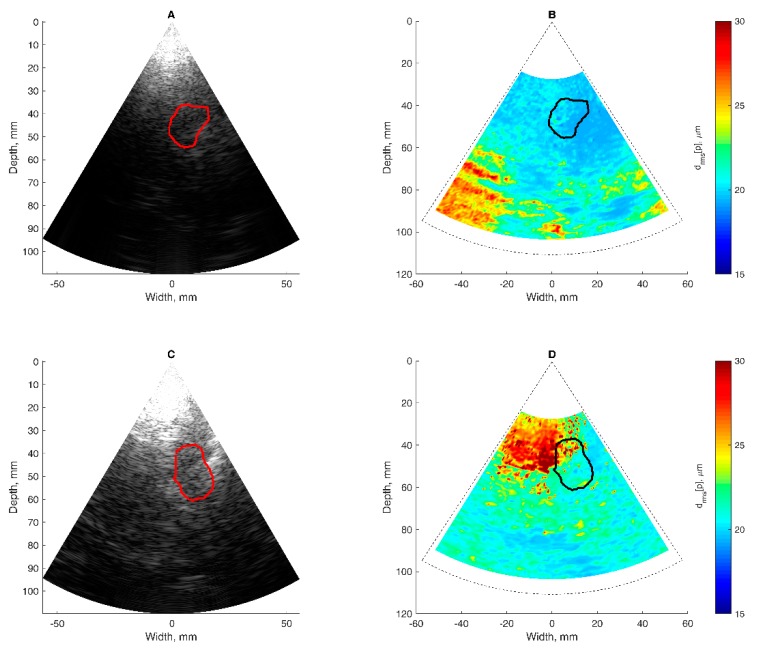
Assessment of endogenous motion in the coronal plane of cranium: (**A**,**C**) B-mode ultrasound image together with the outlined cross-sectional region of the hippocampus; (**B**,**D**) map of endogenous motion in the coronal plane of cranium: root mean square of displacement signal *d_rms_*[*p*] represented in color scale. Hippocampus region outlined with the solid line. Parts (**A**,**B**) correspond to the subject with Alzheimer’s disease, parts (**C**,**D**) to the healthy elderly subject.

Figure 7 presents the analyzed cases, aiming to illustrate the procedure of the displacement’s assessment in the hippocampus region. The Figure 7 illustrations represent data obtained from investigations of a healthy elderly subject and subject with diagnosed Alzheimer’s disease: outlined regions of the hippocampus are presented in Figure 7A–D. Figure 7A,C shows B-scan ultrasound images of the brain obtained by scanning through the temporal window. The cross-section of the hippocampus is outlined by a solid line. The delineation of the area was performed by an experienced neurosonographer. The same outline is provided in a quantitative map of displacement intensities (see Figure 7B,D), calculated using Equation (3). The displacement map in Figure 7B is presented on the same coordinate grid as the B-scans shown in Figure 7A. The original B-scans represent maps of the echogenicity of brain structures, while the displacement maps represent the root mean square of endogenous displacement signals of the brain tissues. The important feature of displacement maps is the possibility to differentiate regions with more stationary structure from pulsating tissues. Some tissue regions of the brain move as they are excited by endogenous sources, while other structures appear relatively more static. Of course, movement intensity depends on the location of blood vessels—the main source of displacement—and also on the anatomical brain region. Relatively static structures found in the displacement map implies an idea of decent management or minimization of the external motions. On the maps shown, some regions outside ROI are marked as having quite a high level of displacements. The question arises if those displacements are confident or related to the artefacts (which is possible in clinical practice). The answer is presented in results Section 3.4, where the confidence of the detected displacements is analyzed.

### 3.3. Region-Averaged Displacements

**Figure 8:**
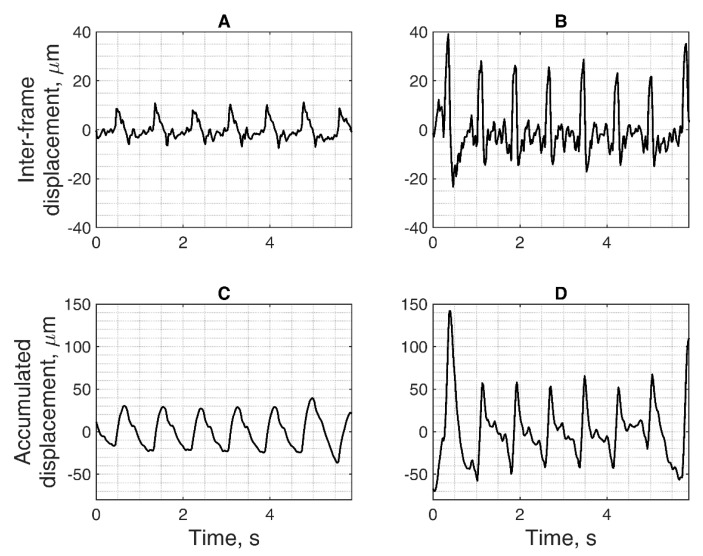
Hippocampus region-averaged displacements signals: (**A**,**B**) inter-frame displacements of the hippocampus; (**C**,**D**) accumulated displacements of the hippocampus. Parts (**A**,**C**) correspond to the subject with Alzheimer’s disease, parts (**B**,**D**) to the healthy elderly subject.

Location (space)-averaged displacements are a potentially useful tool for differential diagnosis of the brain regions. Here, we present an example of displacement-targeted assessment in the specific region of brain structure. For the case of Alzheimer’s disease, the hippocampus is the structure of interest for neurologists. The region of the hippocampus is outlined in Figure 7A. For this region, averaged displacement signal was calculated according to Equation (4). Region-averaged displacement signal is shown in Figure 8. Two forms of displacements are presented: the quantity of displacement detected at instances of a neighbouring B-scan frames, so-called inter-frame displacements (see Figure 8A,B), and another form of displacement in relation to a stationary positioned sonography transducer (see Figure 8C,D), calculated by integration (accumulation) of the inter-frame displacements.

The time-domain representation of signals morphologically resembles the shape of the plethysmogram, as it could be expected from the displacements induced by a quasi-periodic cardiovascular system action. The presented waveforms of brain displacements are of the temporal resolution of 22 ms, so resolving cardiac activity. The quantitative estimate for the ROI mean pulse displacement amplitude (i.e., from negative peak to positive peak) for the subject with Alzheimer’s disease was *d_avg_* = 57.9 ± 26.5 µm; it was *d_avg_* = 107.6 ± 32.0 µm for a healthy elderly participant. Here, average (*d_avg_*) calculated from *Q* = 6 periods for both participants. The amplitude parameter of ROI-averaged displacements of the simplest approach, the other parameters could be implemented as well to explore the diagnostic potential of the proposed technique.

### 3.4. Confident Maps of Brain Displacement

**Figure 9:**
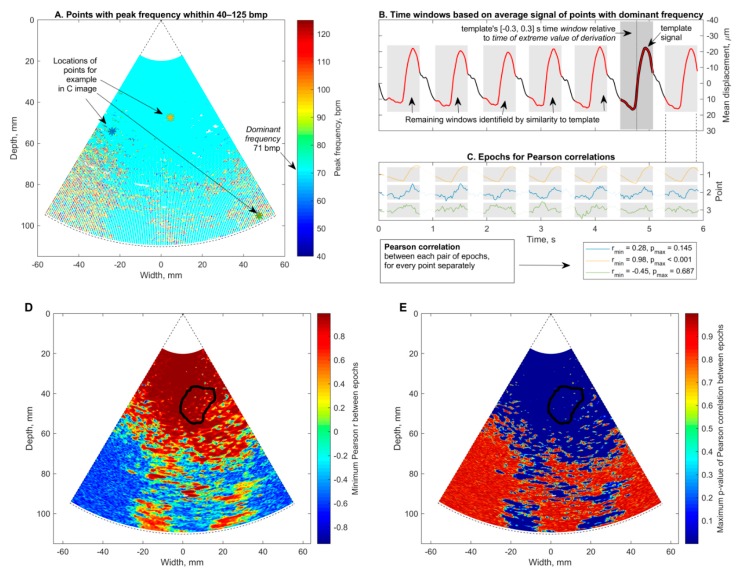
Displacement confidence evaluation by the Pearson correlation of accumulated displacements for a patient with Alzheimer’s disease. (**A**) Dominant frequency (here 71 BPM) determined as the most frequent value of peak frequency from points with spectral peak frequency within a 40–125 BPM interval; (**B**) epochs are time windows of similar waveforms in the average signal of points with dominant frequency; similarity was detected by reference-template matching technique, here seven epochs were identified; (**C**) Pearson correlation was calculated between each pair of signal segments from epochs that belonged to the same spatial point; here 7 × 6:2 = 21 pairs/correlations for every point. The minimum of Pearson correlation r (**D**) and maximum *p*-value (**E**) were used as the confidence criteria—the point was confident if minimum *r* > 0 and maximum *p*-value ≤ 0.01 from all 21 correlations for a particular point. In D and E the cross-sectional region of the hippocampus outlined with black solid line.

Here, we present results of the confident mapping, in which the detected displacements were restricted, employing specificity of endogenous brain tissue motion related to the heart rhythm. US data from the subject with Alzheimer’s disease and healthy elderly participant were analyzed for confidence as examples. Subset *A* was formed from points that satisfied the condition *of f_peak_* ∈ [40, 125] beats per minute (BPM). This subset corresponds to all colored points in Figure 9A. The *dominant frequency f_dmn_* = 71 BPM was determined as the most frequent value in *f_peak_* of points from subset A for both the subject with Alzheimer’s disease and the healthy subject. A new, narrower, subset B was formed only from points that satisfied the condition of *f_peak_ = f_dmn_*; points with 71 BPM are colored sky-blue in Figure 9A. The displacements were averaged only from subset B points according to Equation (4); an example of this averaged signal *d_B_* is depicted in Figure 9B. The template signal *d_temp_* was formed by taking part of the *d_B_* signal that belonged to a [–0.3, 0.3] seconds window. The waveforms similar to *d_temp_* were automatically detected using the normalized cross-correlation function. The time windows of these similar waveforms (i.e., all grey patches in Figure 9B) were identified as epochs. The same epochs were used to split the *d^ftlr^*[*p*] signal into segments (see Figure 8C) for every spatial point separately. Selected points of the confidence map (see blue, orange, and green stars in Figure 9A) have corresponding displacement waveforms (see Figure 9C). The repeatability of these three example waveforms was quantified with a Pearson correlation coefficient ranging from −0.45 to 0.98.

**Figure 10:**
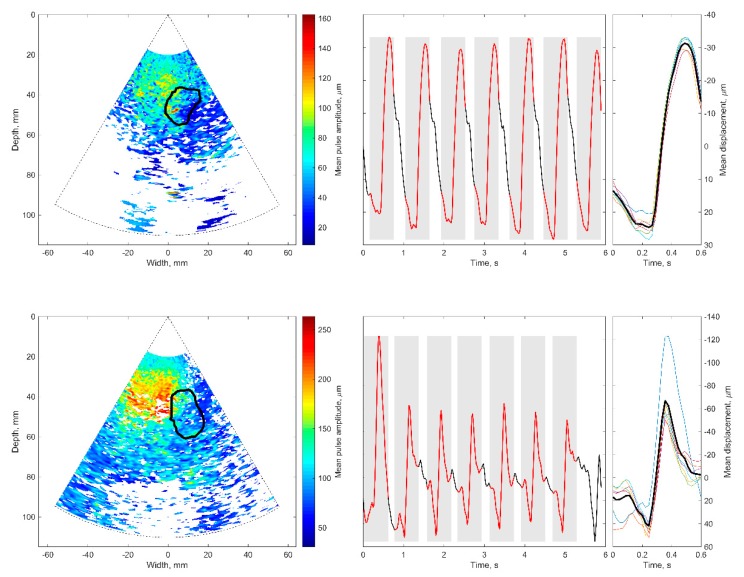
Confidence mapping of displacements in the coronal plane of the brain (**upper** images for a patient with Alzheimer’s disease, **lower** images for healthy elderly participant): (**left**) locations of confident displacements with annotated hippocampus ROI; (**middle**) spatial averaged accumulated displacements in the ROI, similar segments are in grey rectangles with red lines; (**right**) superimposed similar segments (tiny colorful lines) and their average (bold black line).

Then, the thresholding Pearson correlation coefficient further restricted the time variance of waveforms from every spatial point. The waveform repeatability at spatial point *d_ftlr_*[*p*] was assumed to be confident if Pearson *r* > 0 and *p*-value ≤ 0.01 (raw values depicted in Figure 9D,E). The mean pulse displacement amplitude calculated from confident ROI spatial points was *d_avg_* = 58.4 ± 26.3 µm for the patient with Alzheimer’s disease; it was *d_avg_* = 109.4 ± 31.7 µm for the healthy elderly participant. This thresholding made it possible to obtain the final maps of confident in vivo displacements, which are presented in Figure 10.

Only example maps of displacements in particular subjects were included. The discolored areas in the map seen at Figure 10 are not related to cardiovascular pulsing, so it is recommended they are excluded when averaging. The excluded locations appeared at bigger distances from the array transducer or deeper in the intracranial structure. Possibly, the echoscopic signals from deeper structures are more attenuated. At these locations, the unrestricted map (see the lower-left corner in Figure 7B) of in vivo displacements indicates relatively intense movements. However, these movements were quantified as uncorrelated to cardiovascular pulsing and it is recommended they be rejected from further analysis.

Concerning displacement confidence in the ROI of in vivo RF data, the better time repeatability is indicated. The map in Figure 10 depicts almost 100% confident estimates in ROI of the hippocampus, as in the unrestricted map of displacement intensity observed in Figure 7B. This can be explained by the relatively shallow location of ROI and relatively good conditions of transcranial echoscopy.

It was expected that the confidence map of detected displacements could increase the accuracy of spatial averaging in ROI. The assessment suggested excluding points of non-confident displacement from hippocampus ROI. This exclusion of points resulted in the changes in ROI averaged estimates of displacement pulse amplitude by +0.8% in the case of the patient with Alzheimer’s disease and by +1.6% in the case of the healthy elderly participant. It appears that the confidence did not have a strong influence in the accuracy of ROI-averaged estimates in this example. However, confidence analysis helps to differentiate locations on maps of movement intensity. In other words, confidence analysis reveals the spatial locations where motion is caused by undefined source, which is not according to heart pulse. To quantify confidence in motion maps of brain tissue, the intra-subject repeatable cardiovascular pulsing appeared as a feasible source of endogenous displacements.

## 4. Discussion

The technique for quantitative mapping and parameter assessment of brain tissue endogenous displacements of micrometer range was developed and tested on physical phantoms and on in vivo RF ultrasound data. Mainly, our goal was to present technical aspects and potentials of the imaging quantification using raw RF ultrasound signals.

Pre-clinical tests provided by the same hardware and signal processing algorithms as in two in vivo examples showed an acceptable agreement between excited harmonic displacements, realized by dedicated positioning systems. Tests showed acceptable suitability of the proposed hardware and software means for tracking an endogenous-range displacement in the physiological frequency range of heart rate. The detection of harmonic displacements was tested using the motion phantom, where displacements in TMM were induced from the solenoid. Our algorithm enabled the detection of harmonic displacements in the range from 3 to 124 µm peak-to-peak. An in vitro displacement signal was detected successfully for the frequencies and amplitudes, which were close to the detectable in vivo values of the endogenous motion in the presented clinical examples. This indirect pre-clinical estimation of the frequency response and sensitivity of our scanning protocol and algorithm showed that it is sensitive enough for the detection of endogenous movements during a clinical trial.

The estimation of micron-range displacements implied specific technical and methodical difficulties to be concerned about. Elaborating the in vivo scanning protocol, we considered possible external movements induced by the sonographer. Artefacts induced by sonographers working “by hand” are in the range of several tens of microns, while in the case when a dedicated transducer holding system was applied they were found to be only 2 µm peak-to-peak. We noticed that the suitable holder of the transducer is important hardware to also dampen other external movement artefacts of the registered signal, which otherwise can reach a peak-to-peak range from 15 µm to 45 µm, this covering practically all of the endogenous brain tissue displacement range. After experiments, we can conclude that external motions induced by the sonographer, tested subject, and environment seem to be manageable and could be minimized by utilizing the ultrasound probe and subject head holding system.

The obtained high beat-to-beat repeatability of the detected displacements can be hypothesized as a consequence of cardiovascular pulsations in intracranial structures. Those pulsations made it possible to estimate a confidence of displacement mapping in the presence of other disturbances. The intra-subject similarity of periodic displacement waveforms could be expressed by the normalized correlation coefficient, which was more than 0.8. The intra-subject similarity of periodic signal morphology was shown in the hippocampus ROI of the patient with Alzheimer’s disease and in the healthy elderly participant. The confident displacement amplitudes in the entire analyzed B-scan ranged from 8.3 µm to 162.5 µm for the patient with Alzheimer’s disease and from 30.6 µm to 263.2 µm for the healthy participant.

The analysis showed that displacement confidence mapping was not feasible before a spatial averaging in the hippocampus ROI. The restriction to only average confident displacements resulted in only 1.1% underestimation. The good enough confidence of detected displacements in the hippocampus ROI is possibly related to the dense vascular network sourcing strong pulsing in this brain structure. The confidence mapping could be feasible for more deeply-located structures of the brain, such as the midbrain. Limitations of the present study are related to difficulties with micron-range displacement experimentation, the adequacy of an appropriate phantom with well-mimicking ultrasound velocity, and attenuation of the tissue in vivo with a limited frame rate of the scanner, which limits a time resolution. The methodical limitation is the displacement field detection in the direction of US beam propagation only. The obtained longitudinal estimates of accumulated displacements were only in a single projection of the endogenous motion field, which was three-dimensional. The transverse (with respect to scanning lines) component of motion could be estimated by updating the algorithm for detection of two-dimensional displacement vector field. The three-dimensional detection of intracranial motion would require an alternative transcranial scanning window, not only the trans-temporal bone window, which was the only one exploited in the current study. Trans-cranial acoustical access with US waves is limited by the anatomy of the bone of the cranium. So, the alternative acoustic windows would be problematic to obtain a sufficient B-mode image. The main pulsation source in brain tissue is supposedly the blood pulsation in vessels, which causes the pulsation of adjacent tissues. The pulsation in a particular location of tissue depends on the location and strength of the source, degree of damping between the source of vibration and the tissue of interest, and the elasticity of the tissue. Therefore, the other limitation of the present approach is that we could not separate the influence of pulsation damping in intermediary tissue layers from the tissue elasticity (or rigidity) of the registered displacement of particular location of tissue. The displacement in the particular location of tissue depends on the location and strength of the source, degree of damping between the source of vibration and the tissue of interest, and the elasticity of the tissue. Although we were able to register absolute displacements, this limitation made a direct estimation of tissue elasticity distribution problematic. The important feature of displacement maps is the possibility to differentiate regions with more stationary structure from pulsating tissues. Some tissue regions of the brain move as they are excited by endogenous sources, while other structures appear relatively more static. Relatively static structures found in the displacement map imply an idea to use those structures for decent management or minimization of the external motions.

Researchers of magnetic resonance imaging (MRI) approaching functional MRI in the brain are also encountering the problem of endogenous and global motions. Brigth et al. [15] discussed fMRI, which suffers from insufficient temporal resolution. They declared that fMRI output is contaminated with head motion, cardiac pulsations, and other global noise sources. Shirzadi et al. discussed endogenous motions as a second main limitation in their study using MRI [16]. Our RF ultrasound-based study with a temporal resolution of 22 milliseconds showed that is feasible to detect intra-subject repeatable displacement waveforms of brain tissues.

In general, endogenous brain tissue motion has a complex and not completely known pattern, which probably has fast components of heart activity and vascular pulsatility, and also slow wave components induced by respiratory activity and brain autoregulation, other patient-induced artefacts. The definition of motion-inducing sources is beyond this study, but confidence mapping related to slow waves is suitable for further investigations.

Quantitative confident values of the endogenous motion and revealed morphology of displacement signals could open new diagnostic possibilities using conventional scanners with RF output. In summary, the technique described has the potential for quantitative imaging and targeted parametric mapping of brain displacements and could be applied for future clinical trials.

## Supplementary Materials

Supplementary materials can be found at https://www.mdpi.com/2075-4418/10/2/57/s1.
