# Supplementary material for: Quantification of Endogenous Brain Tissue Displacement Imaging by Radiofrequency Ultrasound
Source: Diagnostics (Basel). 2020 Jan 21;10(2):57. doi: 10.3390/diagnostics10020057 (PMC7168898; doi:10.3390/diagnostics10020057)
Supplement: Supplementary file 1 [file diagnostics-10-00057-s001.pdf]

Means of stabilization of array transducer and patient head positioning were elaborated. The transducer holding system on XYZ table is shown in Figure S1A together with positioning of subject head on holders. Stabilizing and easily aligned transducer holder is shown in Figure S1B.

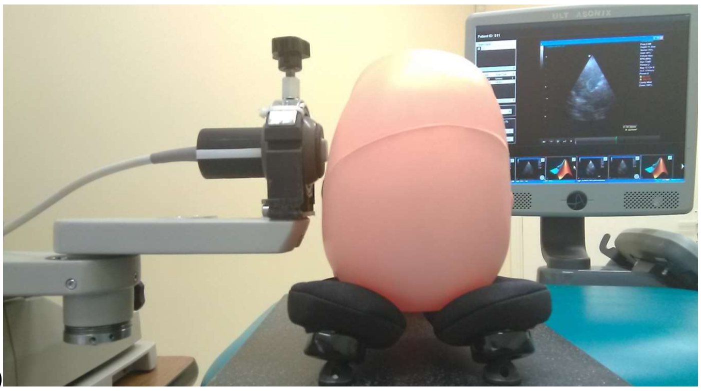

(a)

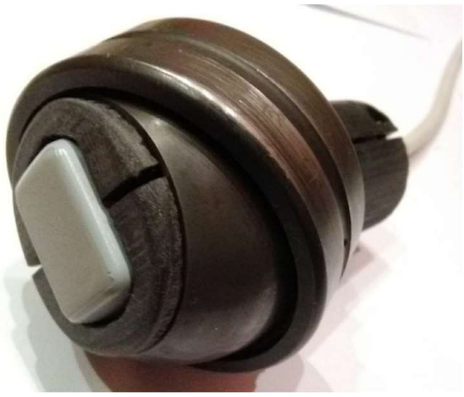

(b)

Figure S1. (**a**) With help of holders' ultrasound probe can be adjusted perpendicularly to the middle temporal bone to obtain the coronal image; (**b**) Setup of phased array transducer holder in spherical bearing.

A spherical plain bearing (model GEG40ES-2RS, from Astbearings.com) is used in the construction of phased array transducer holder. A spherical bearing enables smooth alignment of scanning transducer: for beam direction needed and for scanning plane dislocation. With chosen bearing it is possible to adjust beam direction in the sector angle ± 17°. It is possible to dislocate the scanning plane to any angle in the range of 360°. Dislocation of scanning plane is possible with rotation of phased array around its axis of symmetry. Our transducer has the largest correctional dimension of 36 mm. while the bearing sphere is 60 mm in diameter has a cylindrical hole with a diameter 40 mm. Therefore we used 3D printer to produce a transducer-fitted bushing with an outer diameter of 40 mm to fit cylindrical hole of spherical bearing. The bushing of a cylindrical outer shape was 3D printed split along the axis. The two parts are fixed with bolts when phased array is inserted. The inside shape of the bushing was printed to support and fit the transducer tightly. The bushing with transducer we inserted into the hole of bearing and fixed here with the help of a stud. To the outer diameter (∅68 mm) of the spherical bearing we tuned the holder, so the transducer in a spherical bearing was fixed to the arm of mechanical XYZ table. This table which enables an accurate dislocation in 3 directions we adopted from the fundus camera (CF-60UVi, Canon). With help of XYZ mechanics and a holder the scanning transducer is positioned to the required location on a temporal bone of the head. The position is fixed with the help of brakes of an original Canon XYZ table. XYZ table was fixed to firm, the fundus camera dedicated rack on wheels. With the help of rack on wheels we have a possibility to position transducer to the right or the left side of the head. Head of supine lying subject was supported with the help of dual dub-occipital head support (Model PSO4R/PSO4L from SunriseMedical.com). This head support of two cushions limited possibility to swing head right-left and helps subjects to resist the slight pressure from phased array.

The agarose gell was used as tissue mimicking material (TMM). The background of phantom was manufactured by mixing 5 g/L agar concentration in distilled water. The predicted [11] Young modulus was 7 kPa. To obtain scattering we dispersed 0.3 g of graphite powder in 1000 mL of agar mixture. Wooden rod (2 mm in diameter) was embedded in TMM. The photo of This rod was fixed to solenoid actuator inducing linear motion. The push-pull motion of rod caused the displacements field in agarose gell at defined frequency, what was set by generator.

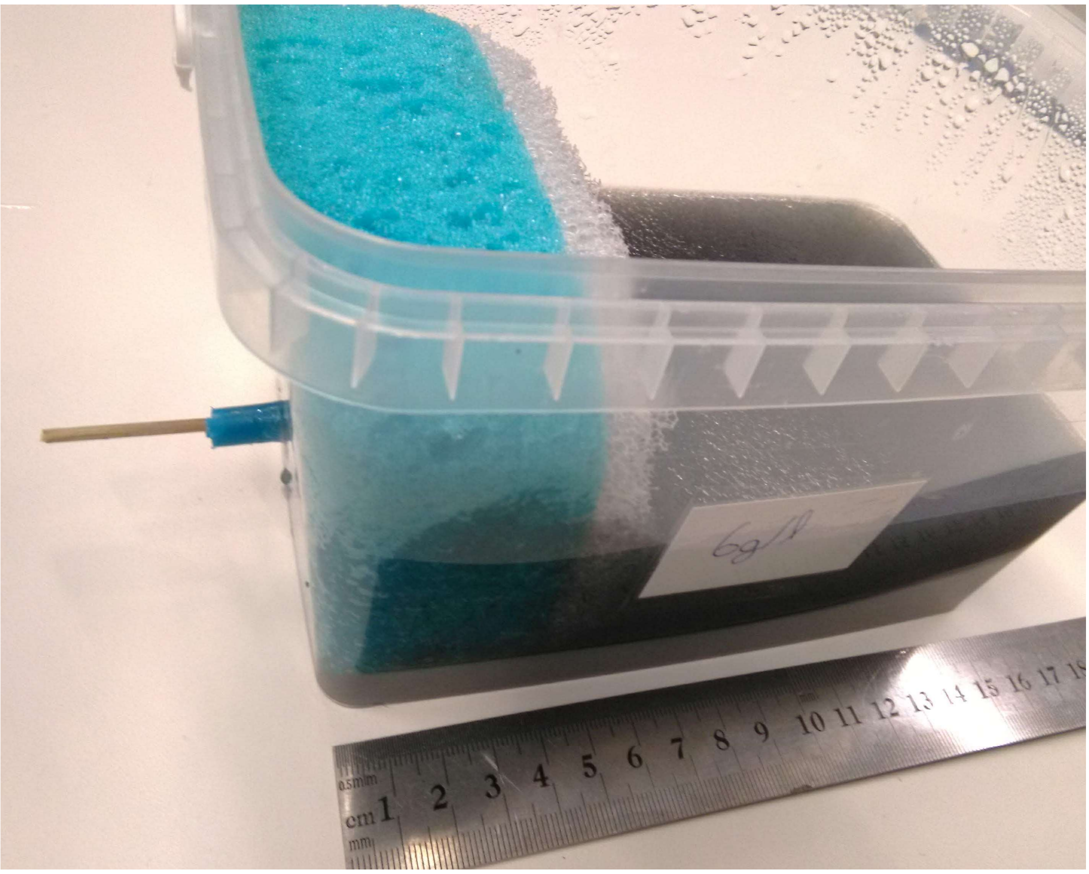

Figure S2. The photo of prepared TMM in the container. Wooden rod (2 mm in diameter) was embedded for more than 9 cm in the TMM trough sidewall of container. The rod was actuated linearly by push-pull motion from solenoid, excited at defined frequency and voltage amplitude from geerator.
